# Outcome of Children with Wilms’ Tumor in Developing Countries

**DOI:** 10.25122/jml-2019-0084

**Published:** 2020

**Authors:** Gholamreza Bahoush, Elahe Saeedi

**Affiliations:** 1. Department of Pediatrics, Ali-Asghar Children Hospital, Faculty of Medicine, Iran University of Medical Sciences, Tehran, Iran

**Keywords:** Wilms’ tumor, overall survival, event-free survival, pathology

## Abstract

Wilms’ tumor is the most common kidney tumor of childhood. The outcome of this malignant tumor has improved due to the improvement of therapeutic strategies. The most important factor in determining the prognosis of these patients is the histopathology subtype of the tumor; unfavorable histopathology is seen in only 11.5% of the patients, which accounts for 52% of deaths. Therefore, the aim of this study was to determine the outcome of children with Wilms’ tumor referred to our hospital over a period of 10 years. This is a retrospective cohort study, and the target population included all patients with Wilms’ tumor referred to Ali Asghar Hospital and were treated according to the National Wilms tumor study 4 (NWTS-4) protocol. All patients’ data were extracted from the medical records of the department. Overall survival and event-free survival (EFS) were analyzed by the Kaplan Mayer method in the SPSS software, version 23. Fifty-two patients (24 male and 28 female patients) with Wilms’ tumor were included. The mean age of the subjects was 40 months. The most common stage among boys and girls was stage II (23.08% and 28.85%, respectively). Our findings revealed that the overall five-year survival of patients was 87±5%; this figure was determined as 100% for boys and 76.8% ± 1.6 for girls (P = 0.018). Our findings show a dramatic improvement in the outcome of children with Wilms’s tumor, and our results are comparable with other results from developed countries. Gender may be an independent prognostic factor of children with Wilms’ tumor.

## Introduction

Wilms' tumor is the most common kidney tumor that accounts for 6% of infants and childhood malignancies [1, 2]. This tumor is also called nephroblastoma because of its renal origin. Earlier, due to the large size of the tumor and little effort in its operation, the mortality rate was high. However, with the advancement of surgical and anesthetic procedures, better caring for children and providing chemotherapy and radiotherapy, survival rates have increased by over 90% [3, 4].

The features of the Wilms tumor are associated with some congenital syndromes and abnormalities, including the WAGR (Wilms' Tumor-Aniridia-Genitourinary Anomalies-Mental Retardation) syndrome, Denys-Drash syndrome, and Beckwith-Weidman syndrome [5, 6].

Wilms' tumor can be unilateral or bilateral. Concurrent cases of bilateral tumors have been attributed to 4.4 to 7% of cases, and asynchronous cases are accounted for 1-1.9%. The mean age of patients with unilateral Wilms' tumor is 28 months in boys and 30 months in girls, and this age range is lower in patients with bilateral Wilms' tumor. In fact, 78% of these cases are seen in children aged 1 to 5 years. The prevalence of this disease is almost identical in both sexes (male to female ratio: 0.9%), and 1% of cases are associated with familial cases [7, 8].

The most common clinical symptoms include abdominal pain, especially in cases of hemorrhage, hematuria, fever, and elevated blood pressure (25% of cases). The lung is the most common metastasis site in Wilms' tumors [9]. Furthermore, histopathology of the tumor is the most important factor in determining these patients' prognosis, where the unfavorable histopathology features are seen in only 11.5% of the patients but account for 52% of deaths [10, 11].

There are different therapeutic protocols for children with Wilms' tumors, and the most important are the SIOP (Societe Internationale D'oncologie Pediatrique) and NWTS (National Wilms Tumor Study-4) protocols. Of course, National Wilms Tumor Study-4 (NWTS-4) was used as a treatment protocol for children with Wilms' tumor in our center. Regarding the high prevalence of this disease among children and the importance of follow-up for a better therapeutic plan, we decided to investigate the survival rate in patients with Wilms' tumor referring to the Ali Asghar Hospital in Tehran for 10 years. We also compared patients' outcomes with other studies' valid results to provide solutions for improving existing therapies.

## Material and Methods

This retrospective cohort study included patients with Wilms' tumor that were referred to Ali Asghar Hospital from Tehran, Iran, from 2005 to 2014. Medical data of all patients, including the age of patients at diagnosis, the date of diagnosis, gender, the type of surgery, pathology, the presence or absence of anaplasia, the presence or absence of metastasis, and the type of metastasis, radiotherapy, recurrence of patients and duration of follow-up after diagnosis were extracted from medical records and recorded in a checklist. All enrolled patients were treated according to the NWTS-4 protocol.

### Data analysis

All enrolled patients, diagnosed and treated between 2005 and 2014, were analyzed for clinical and pathological data, overall- and event-free survival rates. The follow-up duration from the date of diagnosis to death of any causes or last date of follow-up was used to calculate the overall survival rate. We used the follow-up duration from date of diagnosis to either date of relapse or death of any causes (each happened earlier) for calculating the event-free survival rate.

The Kaplan-Meier test was used to determine overall survival (OS) and event-free survival (EFS). Log-Rank method was employed to measure the survival rate, and a P-value lower than 0.05 was considered as significant. Based on the median follow-up (56 months), the five-year OS and EFS values of patients were determined.

### Ethical considerations

Patient information was only available to the executor, and the name of the patient remained confidential. The information remained completely reserved. This project was approved by the Ethics Committee of Iran University of Medical Sciences; approval number: IR.IUMS.FMD.REC 1396.143957.

## Results

A total of 52 patients were studied, of which 24 (46.15%) were boys, and 28 (53.85%) were girls. The mean age of the subjects was 40 months. 77.55% of patients were over 24 months of age. Of the total of 52 patients, 6 died (one patient had stage IV, two had stage III, and three had stage II). The most common causes of death were pulmonary metastases (3 cases), followed by liver, brain and peritoneum metastasis. The most common manifestation of patients at the time of referral was painless abdominal mass, and the most common metastasis sites were the lung and liver.

**Figure 1:**
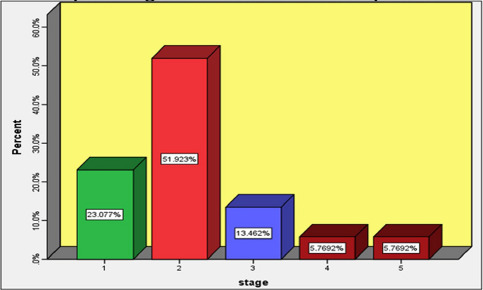
Distribution of tumor staging in the studied patients.

Tumor staging, according to NWTS, is shown in Figure 1. Stage II had the highest incidence (27 patients), followed by stage I (12 patients), stage III (seven patients), stage IV (three patients), and stage V (three patients).

In Table 1, staging distribution is presented based on the two sexes. The most common stage among boys was stage II (23.08%), followed by stage I (9.62%), stage III, and stage V (5.77%), and the lowest incidence belonged to stage IV (1.92%).

**Table 1:** Distribution of tumor staging by gender.

Stage	I	II	III	IV	V	Total
Male	5	12	3	1	3	24
Female	7	15	4	2	0	28

Among the girls, stage II was the most common (28.85%), followed by stage I (13.46%), stage III (7.69%), and stage IV (3.85%). Considering the P-value of 0.42, the distribution of tumor staging was not significant based on gender.

Based on the pathology (Table 2), 17 (32.69%) subjects had unfavorable, and 35 (67.30%) had favorable histopathological features. By age, 60% of subjects under 24 months of age showed favorable status, while 40% had an unfavorable status. On the other hand, 70.27% of patients above 24 months of age showed a favorable status, and 29.72% exhibited an unfavorable status.

**Table 2:** Patients’ pathology based on age and sex.

Pathology	Sex		Age (month)	
	Male	Female	>24	<24
Favorable	8	17	26	9
Unfavorable	6	11	11	6

The pathology’s distribution was determined based on gender as well. The situation was categorized as favorable in 75.00% of cases and unfavorable in 25.00% of cases. Among the girls, the situation was reported as favorable in 60.72% of cases and unfavorable in 39.28% of cases. The difference between the two sexes was not significant (P = 0.21).

**Figure 2:**
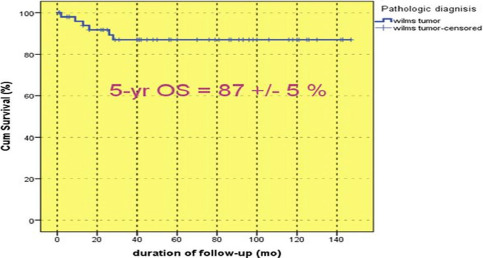
Overall Survival of all enrolled patients.

**Figure 3:**
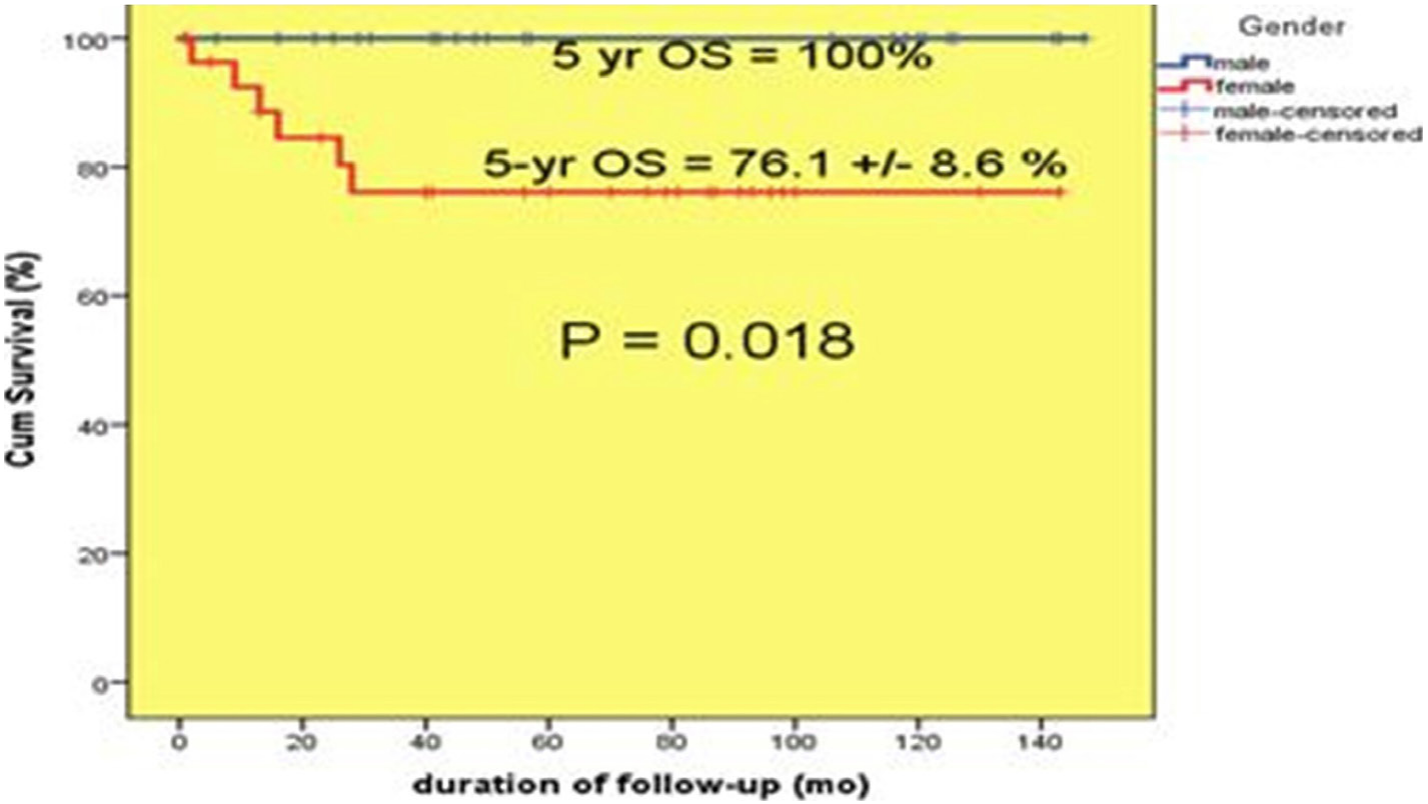
Total survival rate of patients by sex.

Based on the median follow-up months of patients at 56 months, the 5-year overall survival rate of patients was calculated using the SPSS software. The results were as follows: the overall five-year survival of patients was determined as 87±5% (Figure 2). The rate was determined as 100% and 76.8±1.6% for boys and girls, respectively (Figure 3, p=0.018).

Survival rates were significantly better in boys but, as there was no significant difference between the two types of pathological status (favorable or unfavorable), the difference in survival between the two genders is a factor affecting the outcome of Wilms’ tumor. The male gender has a better prognosis than the female gender as an independent factor.

The overall survival rate, based on age in subjects under 24 months of age and above 24 months of age, was calculated as 89.96±0.8% and 85.6±0.9%, respectively. However, this difference was not statistically significant (P = 0.63). Based on the type of pathology (Favorable: 90±9.5% and unfavorable: 86.2±5.8%), the prognosis of patients in the favorable category was better; however, this difference was not statistically significant (P=0.8).

**Figure 4:**
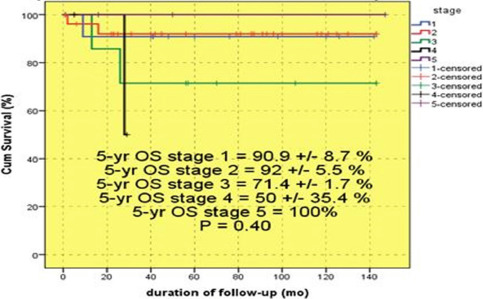
Overall and event-free survival rate of patients based on tumor stage.

The 5-yr OS rates of all enrolled patients were: 90.9±8.7% (stage I); 92±5.5% (stage II); 71.4±1.7% (stage III); 50±35.4% (stage IV). All three patients with stage V were alive during the study (Figure 4).

**Figure 5:**
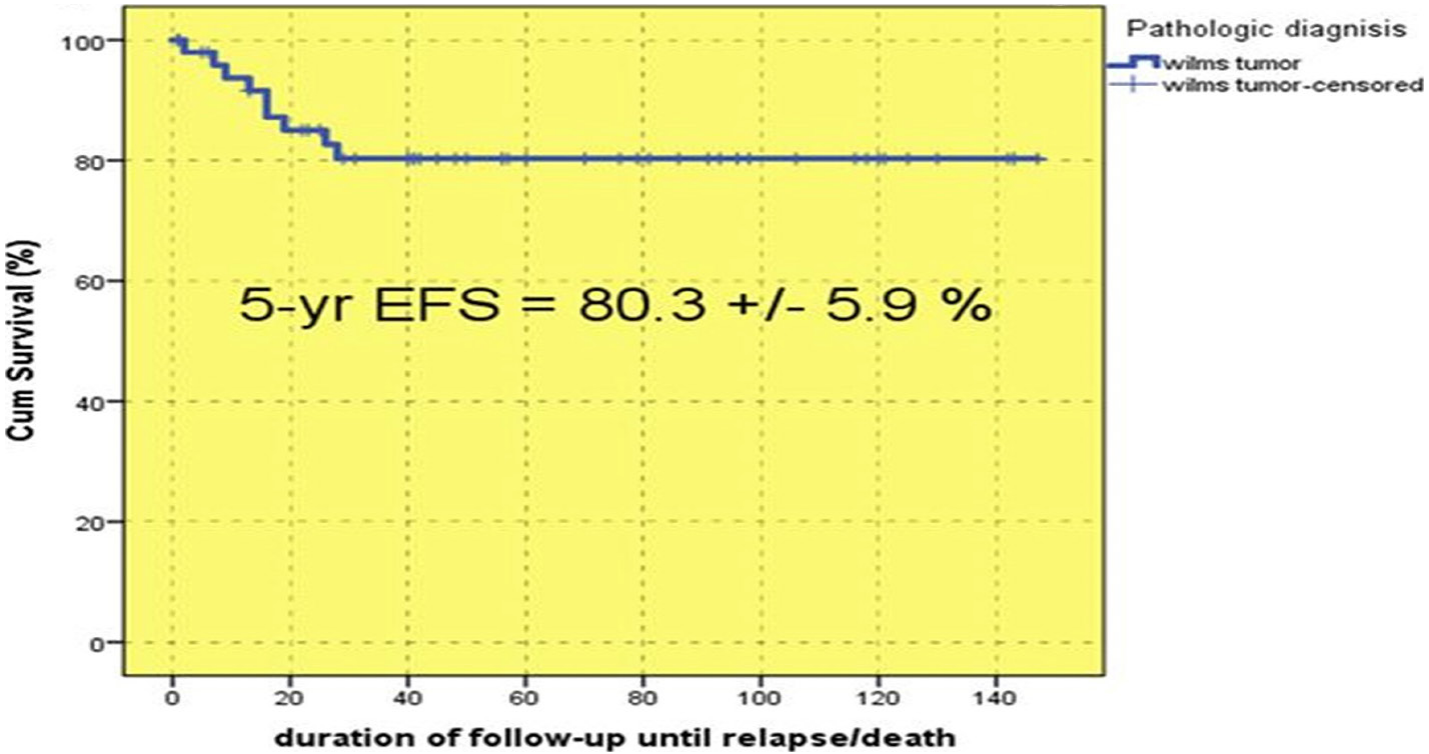
Event-free survival of all enrolled patients.

The 5-year EFS was 80.3±5.9% (Figure 5): 95.2±4.5% in boys and 68.2±9.4 in girls (P = 0.024), indicating a better status among boys.

**Figure 6:**
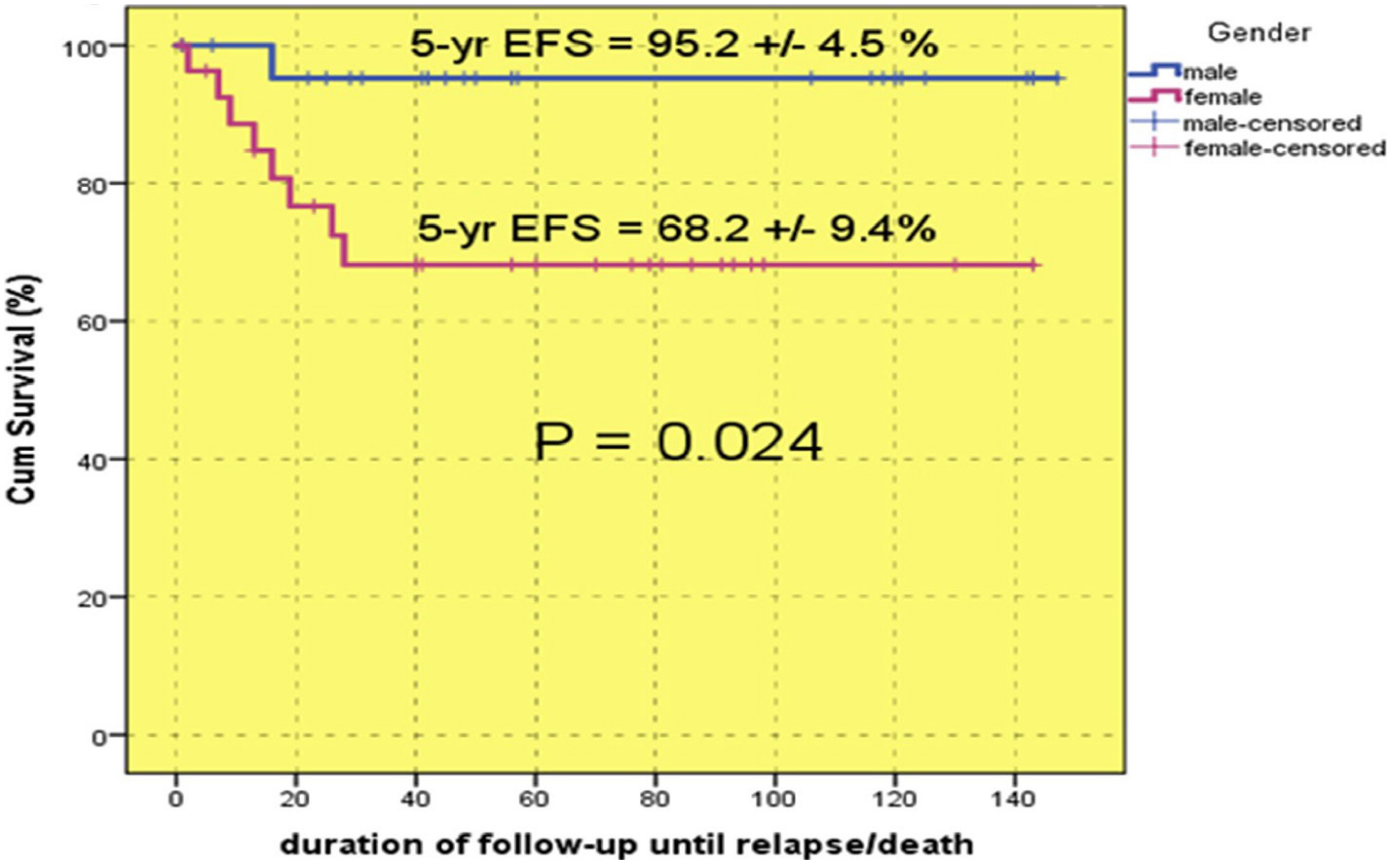
The5 -year EFS.

EFS rates were determined according to the pathology type - 80.3±6.7% in the favorable and 80±1.26% in the unfavorable category, which did not show a considerable difference between the two categories, while the overall survival of the favorable category was better (Figure 6).

EFS was also calculated based on stage (stage I: 90±9.5%; stage II: 83.8± 7.4%; stage III: 71.4±1.7%, stage IV: 50±35.4; stage V: 66.7±27.2%; P = 0.75). Stage IV had the lowest, and stage I had the highest EFS rate.

According to the multiple logistic regression analysis using the Cox regression model, gender was the only risk factor that remained statistically significant and independently effective on the event-free survival of all enrolled patients (P = 0.034). However, this result was not achieved after analyzing the overall survival (P=0.95) of all enrolled patients (Table 3).

**Table 3:** Cox regression multivariate analysis for OS and EFS.

Risk factors multivariate analysis for OS					
	Mean	P value	Odd ratio	95.0% CI for Odds ratio
					Lower	Upper
**Age group**	0.250	0.924	0.917	0.154	5.444
**Gender**	0.462	0.951	0.000003	0.000	1.338
**Histology**	0.673	0.120	6.040	0.626	58.266
**Staging group**	0.731	0.058	0.200	0.038	1.059
**Risk factors multivariate analysis for EFS**					
** **	**Mean**	**P value**	**Odd ratio**	**95.0% CI for Odds ratio**
					**Lower**	**Upper**
**Age group**	0.250	0.833	1.162	0.289	4.676
**Gender**	0.462	0.034	0.170	0.033	0.872
**Histology**	0.673	0.075	4.827	0.853	27.314
**Staging group**	0.731	0.118	0.346	0.091	1.309

## Discussion

Wilms' tumor is the second most common retroperitoneal neoplasm and the most common primary kidney tumor in children [12]. This tumor originates from metanephric blastema cells, which, due to lack of complete differentiation, leads to the formation of a triphasic tumor that includes epithelial cells, stromal cells and blastemal cells. Histologically, it is categorized as “unfavorable” and “favorable” [13]. Histological features are the most decisive prognostic factor [14]. Other effective factors include stage disease, tumor weight, age, evidence of no heterogeneity in chromosomes 1p and 16q, and response rates to treatment [15].

Considering that one of the ways of treating cancer regimens is to determine the survival rate of patients who are being treated, this study examined the survival rate in patients with Wilms' tumor.

In this study, 52 patients (24 males and 28 females) were included in the study and had an average age of 40 months. Staging distribution was evaluated between the two sexes; the most common stage among boys and girls was stage II, without any statistically significant difference between the two genders.

However, the results of the Cox regression model showed that gender might not be considered an independent prognostic factor for the overall survival of children with Wilms' tumor. However, it appears that gender is an independent prognostic factor for their event-free survival.

A study conducted in the UK on 714 patients reported an OS of 87.5%, which is consistent with the current study (OS: = 87±0.5%). The study also noted an EFS of 77.2%, while we obtained a better result (EFS = 80.3±5.9%) [16]. Another study from Kenya evaluated 39 patients with Wilms' tumor with ages ranging from 0 to 16 years (52% female and 48% male) between 2010 and 2012 using the SIOP treatment method. Of these, only 16% had definite Wilms tumor diagnosis before referral. Most were diagnosed as stage I, but none of the patients were determined as stage I. The results of their research were significantly lower than the current study, with a three-year overall survival of only 41% [17].

In another study from North India, 31 patients with Wilms' tumor with an average age of 3 to 4 years (45.2% boys and 54.8% female) were evaluated from 2008 to 2012. The most common complaint was an abdominal mass, and most patients were categorized into stage III (55%). The treatment method used in this country is NWTS. The results of their study are quite similar to the present study [18].

Another study by Yao *et al.* from 2012 included 67 children with Wilms' tumor. Eighteen cases were diagnosed as stage I, 23 as stage II, 20 as stage III, and 6 as stage IV. There were 46 (68.7%) cases of favorable histology and 21 (31.3%) cases of unfavorable (anaplastic) histology. The rates of four-year event-free survival and overall survival were 78.3% and 81%, respectively. The four-year overall survival rate for cases of favorable histology (85.8%) was higher than for those with unfavorable histology (71.4%). The outcomes of patients with Wilms' tumor in their institution were a little poorer than those of the third National Wilms Tumor Study (NWTS-3). Advanced clinical stage at presentation, unfavorable histology, tumor rupture, relapse, delay of radiotherapy due to ignorance, and premature ending of treatment were the important factors that impacted the outcome of the tumor [19].

In a study carried out by Faranoush *et al.*, 175 patients suffering from Wilms' tumor with an average age of 3 years and 8 months (50.3% female and 49.7% male) were studied from 1990 to 2003 and treated by the NWTS-3 protocol. The 5-yr OS was reported as 76%, and the relapse rate was 4.25%. Most of the patients had a recurrence at the origin (52.7%) and lungs, and most of the patients were diagnosed with stage II (35.4%). In this study, the survival rate among boys was 85±4% and 64±7% among girls. Gender was an influential factor in the survival of patients (P = 0.01), as shown by the results of this study [20].

## Conclusion

Our results are similar to those of developed countries, including England, but better than those reported by many developing or underdeveloped countries, such as Nigeria and Kenya, as well as Asian countries (e.g., India and China). Due to the implementation of the NWTS-4 program at Ali Asghar Hospital and a remarkable improvement in the treatment of these patients over the past, the current therapeutic trend is improving.

## Conflict of Interest

The authors declare that there is no conflict of interest.
